# Establishment of a 3D Model to Characterize the Radioresponse of Patient-Derived Glioblastoma Cells

**DOI:** 10.3390/cancers15164051

**Published:** 2023-08-10

**Authors:** Zoe Strand, Finn Schrickel, Sophie Dobiasch, Andreas R. Thomsen, Katja Steiger, Jens Gempt, Bernhard Meyer, Stephanie E. Combs, Daniela Schilling

**Affiliations:** 1 Department of Radiation Oncology, Klinikum Rechts der Isar, Technical University of Munich (TUM), 81675 Munich, Germany; 2 Institute of Radiation Medicine (IRM), Helmholtz Zentrum München, 85764 Neuherberg, Germany; 3 Deutsches Konsortium für Translationale Krebsforschung (DKTK), Partner Site Munich, 80336 Munich, Germany; 4 Department of Radiation Oncology, University Medical Center, University of Freiburg, 79106 Freiburg, Germany; 5 Institute of Pathology, Technical University of Munich (TUM), 81675 Munich, Germany; 6 Comparative Experimental Pathology (CEP), Technical University of Munich (TUM), 81675 Munich, Germany; 7 Department of Neurosurgery, Klinikum Rechts der Isar, Technical University of Munich (TUM), 81675 Munich, Germany

**Keywords:** patient-derived glioblastoma, three-dimensional cell culture, radiosensitivity, radioresponse, DNA damage, spheroids

## Abstract

**Simple Summary:**

Glioblastoma multiforme is an aggressive brain tumor with a poor survival rate despite modern therapeutic options. In this context, advanced preclinical models and the development of new treatments are urgent. Three-dimensional cultures offer new possibilities for understanding the tumor’s biology. They mimic the tumor microenvironment and its complexity, thus reflecting the patients’ neoplasm more closely. We developed a 3D model to analyze the radiation sensitivity in patient-derived glioblastoma cells.

**Abstract:**

Glioblastoma multiforme (GBM) is the most common malignant primary brain tumor in adults. Despite modern, multimodal therapeutic options of surgery, chemotherapy, tumor-treating fields (TTF), and radiotherapy, the 5-year survival is below 10%. In order to develop new therapies, better preclinical models are needed that mimic the complexity of a tumor. In this work, we established a novel three-dimensional (3D) model for patient-derived GBM cell lines. To analyze the volume and growth pattern of primary GBM cells in 3D culture, a CoSeedis^TM^ culture system was used, and radiation sensitivity in comparison to conventional 2D colony formation assay (CFA) was analyzed. Both culture systems revealed a dose-dependent reduction in survival, but the high variance in colony size and shape prevented reliable evaluation of the 2D cultures. In contrast, the size of 3D spheroids could be measured accurately. Immunostaining of spheroids grown in the 3D culture system showed an increase in the DNA double-strand-break marker γH2AX one hour after irradiation. After 24 h, a decrease in DNA damage was observed, indicating active repair mechanisms. In summary, this new translational 3D model may better reflect the tumor complexity and be useful for analyzing the growth, radiosensitivity, and DNA repair of patient-derived GBM cells.

## 1. Introduction

Glioblastoma (GBM) is the most common primary malignant brain tumor in adults. The current standard therapy consists of surgical resection, chemotherapy, tumor-treating fields (TTF), and radiation treatment [1,2,3]. Despite this multimodal and aggressive treatment, the rate of tumor relapse is tremendous, and the survival is still very poor. The median survival after initial diagnosis is only 15 months, and the 5-year survival rate is below 10% [4,5].

The malignancy of the tumor relies on its infiltrative nature, resistance to chemo- and radiotherapy, and intratumoral heterogeneity [6]. A functioning DNA damage repair and response is a critical factor for the survival of cells after radiotherapy. Cancer cells often have a reduced ability for DNA repair, making radiation treatment more effective and protecting healthy tissue. On the other side, a heightened DNA repair can render tumor cells more resistant to therapy [7,8]. Other known factors for radioresistance are hypoxic regions within the tumor which leads to less free radicals of the reactive oxygen species and, as a result, less DNA damage caused in this area [9,10,11]. Hypoxia also enhances the presence of cancer stem cells (CSCs), which are thought to be one of the contributing factors for therapy resistance in GBM [12,13,14]. The heterogeneity of the tumor leads to a wide range of cells with different metabolic or genetic adaptations [6,15]. This increases the probability of developing intratumoral resistance to radiation by clonal selection.

Cultivation and characterization of patient-derived GBM cells could facilitate the individualized study of this cancer type for developing novel therapies and may help to predict patients’ response to chemo- and radiation therapy [16]. As a key requirement, reliable preclinical models are needed that can recapitulate the clinical scenario and mimic the properties of the tumor. But commonly used models are mainly too simplistic for this very complex tumor type [17].

In conventional two-dimensional (2D) cell culture systems, monoclonal tumor cell lines grow in a homogeneous monoculture. This may not reflect the architecture of the patient’s tumors [18]. Additionally, it is also still debated how the stiffness of the surface affects the invasion ability of brain tumor cells. Research suggests that rigidity can alter cell morphology and motility [19,20]. Another limitation of 2D culture systems is the uniform distribution of oxygen (usually 20% in incubators) and an unphysiologically high glucose content in cell culture media. While in vivo, tumor cells exhibit a gradient from the periphery to the center regarding oxygen concentration, pH, and nutrient supply. This can lead to different gene expressions and metabolic adaptations of cancer cells [21,22].

In the last few years, the development of three-dimensional (3D) cell culture models has rapidly expanded [23]. There is now a wide variety of different methods and strategies. Many 3D cultures grow as spheroids, usually derived from monoclonal cancer cell lines. After the cells self-assemble in a suspension, they continue to proliferate and increase in volume [24,25]. The basic principle in forming spheroids is to prevent contact with the surface so that the cells are forced to form contact with each other. The cell interactions and the morphology, together with the differing availability of oxygen and nutrients within the spheroids, are one step closer to resembling the in vivo tumors microenvironment [23,26]. For these reasons, tumor spheroids are very useful for analyzing radiation responses in cancer cells [27]. Therefore, 3D spheroids provide a special opportunity to investigate the effectiveness of radiotherapy, which is an important part of the therapy scheme for GBM patients. Furthermore, 3D spheroids promote a deeper understanding of the mechanisms that cause radioresistance of GBM cells.

In order to establish a reliable 3D model of patient-derived GBM cells for analysis of long-term tumor growth and radiation response, we used a conical agarose 3D microwell system [28,29]. The microwells are made of agarose, which allows the diffusion of molecules, and the non-adhesive characteristic prevents cells from attaching to the surface. Using this 3D cell culture model, the success of treatment and biological characteristics can be tested in a more complex and realistic environment. It allows a better understanding of how radiation affects tumor growth and cellular behavior in more natural conditions leading to a translational approach.

## 2. Materials and Methods

### 2.1. Culture of the Glioblastoma Cell Line LN229

The established GBM cell line LN229 was cultivated in Dulbecco’s Modified Eagle’s Medium with 10% FCS and 1% penicillin/streptomycin in a humidified atmosphere with 95% H_2_O, 5% CO_2_, and 37 °C.

### 2.2. Isolation and Cultivation of Patient-Derived Glioblastoma Cells

To generate primary GBM cell lines, the patient-derived tumor tissue was cut into small pieces, transferred into a 6-well plate, and covered with medium-filled agarose cups (provided by Dr. Andreas Thomsen, University of Freiburg [28]). After 1–2 weeks of incubation, the cells were transferred to culture flasks and cultivated in RPMI 1640 medium supplemented with 10% FCS and 1% penicillin/streptomycin at 37 °C, 5% CO_2_, and 95% humidity. In this study, three patient-derived GBM cell lines were included.

Table 1 shows the clinical data of the patients from whom the primary cell lines were derived. All subjects gave their informed consent for inclusion before they participated in the experimental RadGlio study [30,31] that was approved by the Ethics Committee of the Technical University of Munich (TUM) (394/16S).

### 2.3. Irradiation

Irradiation was performed with the RS225A X-ray device (Xstrahl Ltd., Walsall, UK) at a dose rate of 1 Gy/1.07 min (15 mA, 200 kV).

### 2.4. Two-Dimensional Colony Formation Assay (2D CFA)

The cells were seeded in 12-well plates and irradiated 24 h later with 0, 2, 4, 6, and 8 Gy. After two weeks, the cells were washed with PBS, fixed with −20 °C methanol, stained with 0.1% crystal violet, and scanned with the GelCount^TM^ (Oxford Optronix, Abingdon, UK). The colonies were counted with the GelCount^TM^ software version 1.1.8.0 with a minimum limit of 50 cells per colony. The survival fraction (SF) was calculated by dividing the plating efficiency (PE) at the different doses by the plating efficiency at 0 Gy. The survival data were fitted to the following linear model (corresponding to the linear quadratic model with the quadratic term set to 0). The survival fraction SF is described by the radiation dose D and the linear coefficient α:

(1) $$\mathrm{SF}={exp}^{\left(-\alpha D\right)}$$

### 2.5. Three-Dimensional CoSeedis^TM^ Glioblastoma Cell Culture

Three-dimensional CoSeedis^TM^ (abc biopply, Solothurn, Switzerland) matrices (1 × 1 or 2 × 2 mm with 880 or 200 microcavities) were put into 6-well plates and equilibrated for at least 2 h in a humidified incubator at 37 °C with RPMI medium. The cells were detached with accutase, passed through a cell strainer (40 μm), and counted. The cell number per cavity was chosen depending on the matrix size and the experiment. The equilibration medium was removed, and the cell suspension was applied to each matrix. After 20 min, the plates were placed in the incubator allowing the cells to settle in the cavities. The next day the matrices were transferred into new 6-well plates to minimize cell growth on the surface of the plate. The medium was exchanged once per week.

### 2.6. Volumetric Analysis of the 3D Cell Culture

To analyze the volume of the spheroids over time, an image was taken with the GelCount^TM^ (Oxford Optronix, Abingdon, UK) every week. The images were then analyzed with the software ImageJ 1.48v. With an image resolution of 1200 dpi (corresponds to 1200 pixels in one inch), the scale was set at 1 inch = 25.4 mm = 25,400 μm. The measured area A was determined by a greyscale threshold. The minimum particle size was adjusted to exclude dust and artifacts. As the cells aggregated in the cavities as spheres, the volume V was calculated with the following formula:

(2) $$V=\frac{4\pi }{3}{\left(\sqrt{\frac{A}{\pi }}\right)}^{3}$$

### 2.7. Three-Dimensional Colony Formation Assay (3D CFA)

A total of 10 cells/microcavity were seeded in the 3D CoSeedis^TM^ 1 × 1 mm matrices in RPMI 1640 medium supplemented with 25 mM Hepes and 10% FCS according to the protocol for experiments with low cell densities. After 24 h, the cells were irradiated with 0, 2, 4, 6, and 8 Gy. To prevent precipitates, 25 mM Hepes was added to the medium, and the medium was changed every week. Weekly, images were taken with a high-resolution scanner (GelCount^TM^), and the volume was analyzed. The survival analysis was performed after 4 weeks of incubation with ImageJ software. The colonies were identified by a greyscale threshold. Colonies were counted with a binary readout. The 1 × 1 mm matrix consists of 880 cavities; therefore, the number of the developed colonies for each irradiation dose was divided by 880. The survival curves were fitted to the linear–quadratic model (LQM). The survival fraction SF is described by the radiation dose D, the linear coefficient α, and the quadratic coefficient β:

(3) $$\mathrm{SF}={exp}^{\left(-\alpha D-\beta D^{2}\right)}$$

### 2.8. Hematoxylin and Eosin (H&E) and Immunohistochemical (IHC) Staining

The cells were seeded into the 3D CoSeedis^TM^ 2 × 2 mm matrices with 1000 cells/microcavity in RPMI 1640 medium supplemented with 2.5% FCS according to the protocol for experiments with high cell densities. The medium was changed each week. The spheroids were fixed at the end of the experiments as indicated in 4% paraformaldehyde for 6 h and then transferred into 70% ethanol. The paraffin-embedding, slicing, and IHC staining for γH2AX (phospho-histone H2A.X antibody (Ser139), 9718S, Cell Signaling Technology (Danvers, MA, USA), dilution: 1:500), Ki67 (anti-Ki67 antibody [SP6], ab166667, Abcam (Cambridge, UK), dilution: 1:50) and GFAP (anti-glial fibrillary acidic protein antibody, G6171, Sigma-Aldrich (St. Louis, MO, USA), dilution 1:400) was performed by the Comparative Experimental Pathology, Institute of Pathology (Technical University of Munich) using a Bond Rxm (Leica Biosystems, Wetzlar, Germany) with a Polymer Refine Detection Kit. All slides were digitalized using a slide scanner system (AT2, Leica Biosystems). The γH2AX-stained spheroids were analyzed using the software QuPath (open source) [32]. The software differentiates between positive and negative stained cells and calculates the percentage of each proportion. The mean percentage of positively stained cells in the different spheroids was determined for each irradiation dose.

### 2.9. Statistics

The software GraphPad Prism (GraphPad Software, San Diego, CA, USA) was used for the statistical analysis. For the comparison of the cell lines, a *t*-test and two-way ANOVA (analysis of variance) were applied. A *p*-value of ≤ 0.05 was considered to be statistically significant.

## 3. Results

### 3.1. Radiosensitivity of Primary GBM Cell Lines Determined by CFA

**Figure 1:**
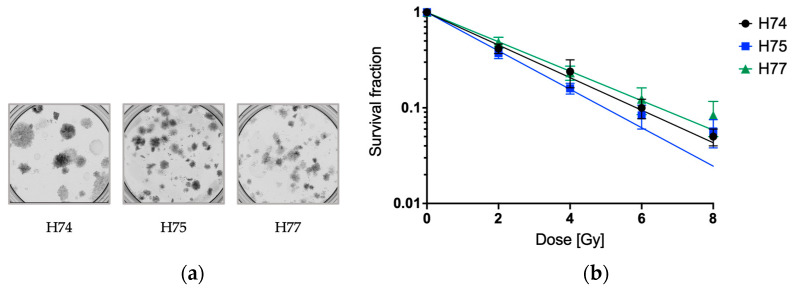
Radiosensitivity of the primary GBM cell lines (H74, H75, and H77) determined by 2D CFA. (**a**) Representative images of the 2D colony formation assay of the primary GBM cell lines (H74, H75, and H77). The images show the spreading growth of unirradiated 2D cultures. (**b**) One day after seeding, the cells were irradiated with 0, 2, 4, 6, and 8 Gy. The plating efficiencies at 0 Gy were 13% for H74, 30% for H75, and 17% for H77. The survival fractions were fitted with the model SF = exp ^(−αD)^. Error bars indicate the SEM of three independent experiments. The *t*-test and two-way ANOVA revealed no significant differences between the cell lines.

To analyze the radiosensitivity of patient-derived primary GBM cells, a clonogenic assay—the gold standard for the determination of radiosensitivity—was performed. As the primary cell lines showed a heterogeneous and spreading growth pattern and hence formed no compact but rather widespread patchy and overlapping colonies (Figure 1a), counting the colonies was difficult and therefore performed manually. A fit of the survival fractions with the LQM revealed unexpected results; no shoulder and negative β values were observed. Therefore, the survival curves were fitted with a linear model (quadratic term β set to 0) (Figure 1b). A dose-dependent decrease in the survival, but no differences between the three primary cell lines, was observed.

### 3.2. Establishment of a 3D Cell Culture Model for Primary GBM

Three-dimensional assays constitute an attractive alternative to 2D assays as they reflect better the in vivo situation. The 3D CoSeedis™ assay (abc biopply) was selected as it offers a novel 3D approach to the traditional CFA. It consists of a conical agarose-based microwell array that allows the growth of up to 880 spheroids in one matrix.

#### 3.2.1. Volumetric Analysis

**Figure 2:**
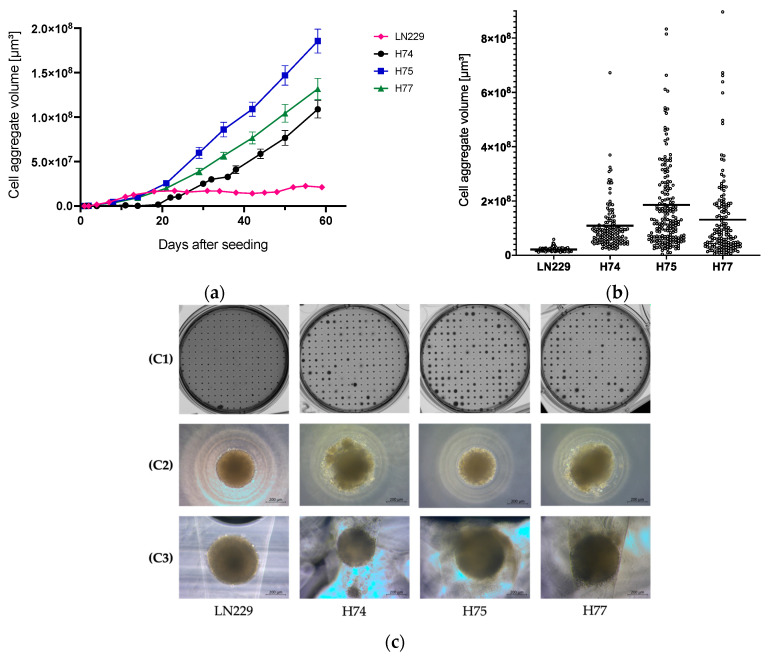
(**a**) Time-dependent 3D growth of the primary GBM cell lines (H74, H75, and H77) and the established GBM cell line LN229. A total of 500 cells per microcavity were seeded in RPMI 1640 supplemented with 2.5% FCS in 2 × 2 mm 3D CoSeedis™ matrices. Each data point represents the mean volume of at least 150 spheroids. Error bars indicate the SEM. (**b**) Size distribution of the spheroids of the primary GBM cell lines (H74, H75, and H77) and the established GBM cell line LN229. The volume of 3D cell aggregates after 60 days was measured and displayed as a scatter plot. Each point represents the measured volume of one spheroid. The mean value is shown as a horizontal line. (**c**) Representative images of the spheroids: (**C1**) shows images of the 3D matrices taken with a high-resolution scanner. The light microscope pictures (**C2**) and (**C3**) were taken at 10× magnification. (**C2**) shows the spheroids from above. In (**C3**), the 3D CoSeedis^TM^ agarose matrices were vertically cut.

To monitor the 3D growth of the primary GBM cell lines (H74, H75, H77) in comparison to the established GBM cell line LN229, images were taken once a week, and the volume of ~200 spheroids was measured. The time-dependent development of the volume is displayed in Figure 2a. The established cell line LN229 showed the highest volume after 2 weeks before the growth started to stagnate. The primary cell lines H74, H75, and H77 showed steady growth over time with a maximum volume of 1.0–1.8 × 10^8^ μm^3^ after 60 days. In comparison to the primary cells, the established cell line LN229 only reached a volume of 2.1 × 10^7^ μm^3^ after 60 days.

To illustrate the heterogeneity of the cell aggregates, the size distribution of the individual spheroids is depicted in Figure 2b. Each point represents the measured volume of one cell aggregate after 60 days of incubation. The associated images of the 3D cultures after 60 days are displayed in Figure 2c(C1). The primary GBM cell lines showed a heterogeneous growth pattern with a larger distribution in volume compared to the established cell line LN229 which revealed a relatively uniform size. This can also be seen in the microscopic images where the spheroids of LN229 exhibited a more homogeneous shape compared to the primary cell lines (Figure 2c(C2,C3)).

#### 3.2.2. H&E, GFAP, and Ki67 Staining

**Figure 3:**
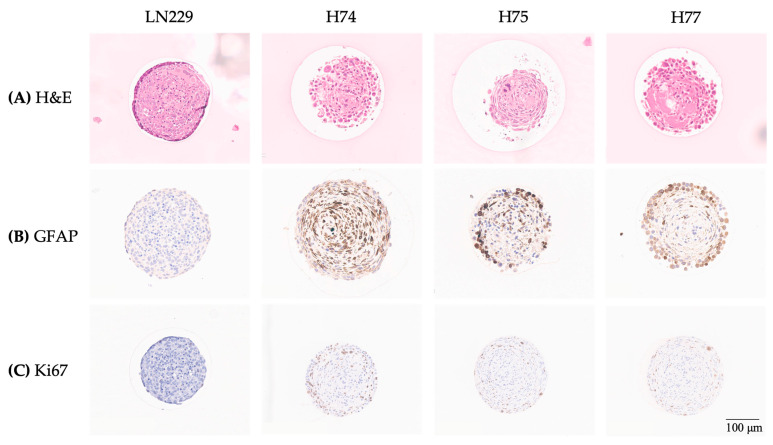
Histology of the 3D cell cultures: Shown are representative images from the established GBM cell line LN229 and the primary cell lines H74, H75, and H77 stained with H&E (**A**), the immunohistochemical detection of GFAP (**B**), and the marker for cell proliferation Ki67 (**C**). The black arrow indicates possible necrotic areas in the spheroid.

For a morphological overview, the spheroids were grown in 2 × 2 mm 3D CoSeedis™ matrices, fixed, and stained with H&E (Figure 3). The established cell line LN229 revealed homogeneous H&E staining and spheroids in a round shape. The H&E staining of spheroids derived from primary cell lines demonstrated a more heterogeneous growth pattern with possibly necrotic areas (black arrow). To validate the origin as glial cells, the expression of the glioblastoma marker glial fibrillary acidic protein (GFAP) was detected by IHC. All three primary cell lines showed a strong GFAP staining, whereas the established LN229 cell line revealed only a weak GFAP staining. The Ki67 staining indicated an enhanced proliferation in the periphery of the spheroids. LN229 spheroids appear to have less Ki67-positive cells compared to the primary cell lines.

### 3.3. Three-Dimensional Colony Formation Assays (3D CFA)

To investigate the radiosensitivity of primary GBM cell lines in the 3D CFA, the 1 × 1 mm 3D CoSeedis^TM^ matrix containing 880 microcavities was chosen as it enables the analysis of up to 880 spheroids for each irradiation dose. To simulate the conditions of the 2D CFA, only 10 cells per microcavity were seeded, and 24 h later, the cells were irradiated with 0, 2, 4, 6, and 8 Gy.

**Figure 4:**
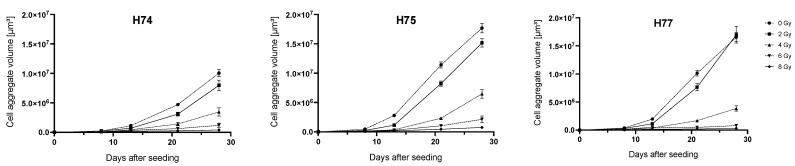
Growth curves of irradiated primary GBM cell lines in 3D culture. A total of 10 cells per microcavity were seeded in RPMI 1640 supplemented with 10% FCS in 1 × 1 mm 3D CoSeedis™ matrices and 24 h later irradiated with 0, 2, 4, 6, and 8 Gy. The volume of the spheroids was determined weekly for each irradiation dose. Mean values and SEM of the spheroids grown in the cavities are shown.

The volumetric growth of the primary cell lines in the 3D CFA experiment was measured weekly for each irradiation dose. As expected, a dose-dependent decrease in the volume with higher irradiation doses was observed in all primary GBM cell lines (Figure 4). The primary cell line H74 exhibited a smaller volume compared to H75 and H77.

**Figure 5:**
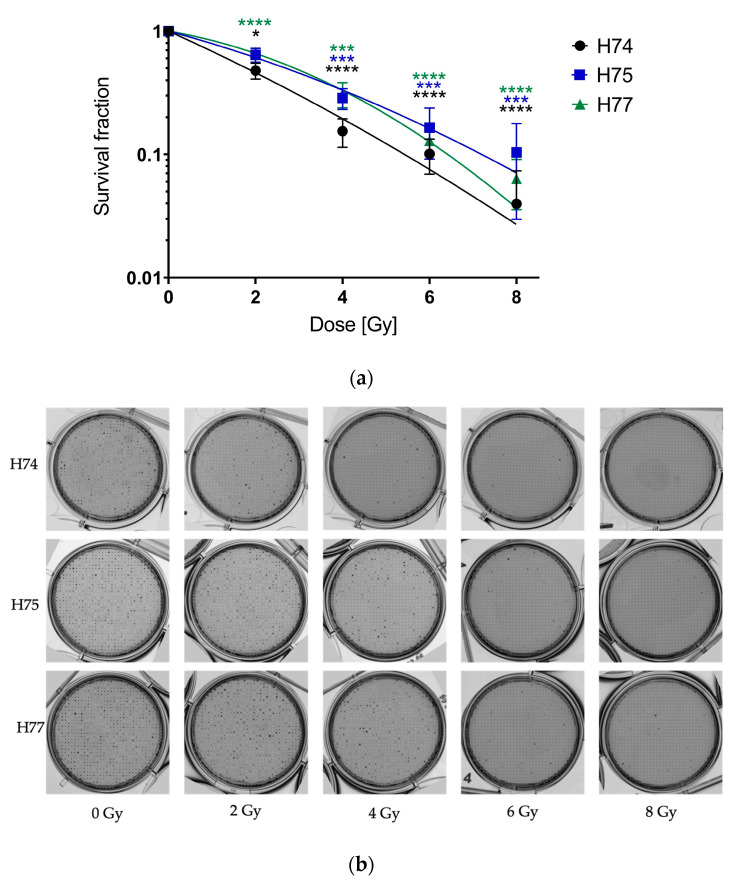
Radiosensitivity of the primary GBM cell lines in 3D CFA experiments. A total of 10 cells per microcavity were seeded in RPMI 1640 supplemented with 10% FCS in 1 × 1 mm 3D CoSeedis™ matrices and 24 h later irradiated with 0, 2, 4, 6, and 8 Gy. (**a**) The survival fraction was determined 4 weeks after irradiation by a binary read-out. For each dose, one 3D CoSeedis^TM^ matrix with 880 possible colonies was evaluated. The survival curves were fitted to the LQM. Error bars indicate the SEM of three independent experiments. The unpaired two-tailed *t*-test and two-way ANOVA showed no significant differences between the cell lines. Significant differences between the survival fraction at the different radiation doses and 0 Gy for each cell line are indicated (* *p* ≤ 0.05, *** *p* ≤ 0.001, **** *p* ≤ 0.0001). (**b**) Associated images of the 3D CFA experiment. The images show the 3D cultures after 28 days of incubation for each irradiation dose.

To determine the survival fraction in the 3D CFA analogous to the 2D CFA, the ability to form 3D colonies in the matrices was evaluated 4 weeks after irradiation by a binary read-out. The number of microcavities containing a spheroid was divided by the number of evaluated microcavities for each irradiation dose. The respective survival data were fitted according to the LQM. In contrast to the 2D CFA, a fit of the 3D survival data with the LQM was feasible, showed positive α- and β-values and hence the survival curve revealed the expected shoulder (Figure 5a). The radiobiological parameters are depicted in Table 2. In line with the 2D CFA, all three cell lines showed a dose-dependent survival with no significant differences between cell lines.

### 3.4. Analysis of DNA Damage and Repair after Irradiation of 3D Cultures

**Figure 6:**
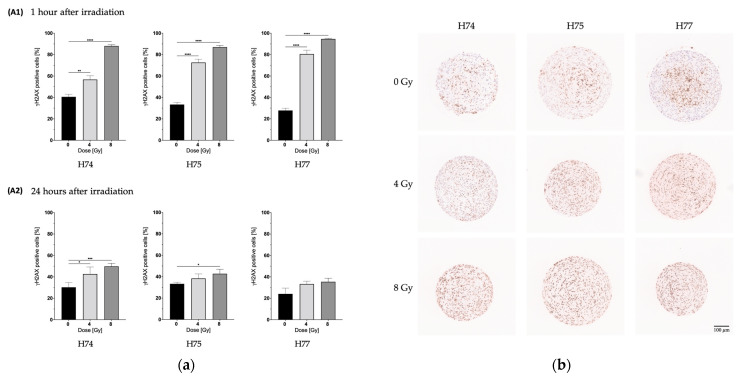
DNA damage after irradiation of primary GBM cells in 3D culture. A total of 1000 cells per microcavity were seeded in 2 × 2 mm 3D CoSeedis™ matrices and 5 weeks later irradiated with 0, 4, and 8 Gy. (**a**) Spheroids were fixed (**A1**) 1 h and (**A2**) 24 h after irradiation and DNA double-strand breaks were analyzed with the marker γH2AX. The results are the means of at least 30 sections from 3 independent experiments. Error bars indicate SEM. Significance was calculated by applying an unpaired two-tailed *t*-test (* *p* ≤ 0.05, ** *p* ≤ 0.01, *** *p* ≤ 0.001, **** *p* ≤ 0.0001). (**b**) Representative images of the γH2AX staining of primary GBM cells in 3D culture 1 h after irradiation.

To analyze DNA damage and repair after irradiation, the primary GBM cell lines were seeded into 2 × 2 mm 3D CoSeedis™ matrices, grown for 5 weeks, and irradiated with 0, 4, and 8 Gy. Spheroids were fixed 1 h and 24 h after irradiation, embedded, cut and stained against γH2AX, a marker for DNA double-strand breaks (DSBs). Representative images of the γH2AX-stained spheroids 1 h after irradiation can be seen in Figure 6b. In the non-irradiated spheroids, the γH2AX signal is enhanced in the center. Irradiation with 4 and 8 Gy uniformly increased the γH2AX staining in all three cell lines.

The percentage of γH2AX-positive cells in the spheroids 1 h and 24 h after irradiation has been quantified and depicted in Figure 6a. Irradiation significantly increased the percentage of γH2AX-positive H74, H75, and H77 cells 1 h after irradiation. As expected, a reduction in DNA damage can be observed 24 h after irradiation. H74 had the highest percentage of γH2AX-positive cells 24 h after irradiation with 8 Gy in comparison to H75, which had a low increase, and H77, which showed no significant increase at all. Only H74 showed a significant increase in γH2AX-positive cells 24 h after irradiation with 4 Gy. A similar trend has been observed for the radiobiological data derived from the 3D CFA, exhibiting lower D_50_ and D_10_ values in H74 in comparison to H75 and H77 (Table 2).

## 4. Discussion

Glioblastomas are heterogeneous and infiltrative tumors that have the ability to adapt and build resistance against existing treatments. Although novel therapeutic approaches are available for other cancer types, treatment options for this complex tumor are lagging significantly behind. Thus, preclinical models that recapitulate GBM pathophysiology and predict later clinical efficiency are one of the key challenges to accelerate progress to successful personalized treatment [33,34]. In contrast to established cell lines that have adapted to their artificial environment, patient-derived GBM cancer cells mirror the original patient’s tumor and can form tumors consisting of a mix of cytologically heterogeneous cells which exhibit unique genetic mutations [35].

In recent years new strategies for the development of 3D models have been implemented [23,36] as these models can mimic the real in vivo conditions in the tumor closer than the conventional 2D cultures [26,37]. On the other hand, 3D cultures can be expensive and time-consuming. Due to their complexity, standardization can be challenging, causing difficulties in reproducibility and comparability [26,38,39].

In this study, the 3D CoSeedis^TM^ agarose-based system was used to culture patient-derived glioblastomas in 3D culture. This system has the advantage of being non-adhesive, so the cells can self-assemble and form a 3D culture [29].

To establish the 3D model with patient-derived GBM and to analyze the best growth conditions, the primary cells, as well as the established cell line LN229 were cultured for 60 days, and the volume of the spheroids was determined (Figure 2a). The different primary cell lines showed a much wider size distribution compared to the established glioblastoma cell line LN299. This indicates a more heterogeneous composition of cells in the spheroids. This can also be seen in the microscopic images where the spheroids of LN229 seem to exhibit a more homogeneous shape compared to the primary cultures (Figure 2c). The heterogeneous size of the spheroids in the CoSeedis^TM^ 3D cultures can reflect the physiological growth patterns of glioblastoma tumors. This culture method offers a great opportunity for patient-derived primary cancer cells. Not only can the spheroid volume analysis of the 3D CoSeedis^TM^ cultures present a higher resemblance to the original tumor, but it also can simplify the subsequent evaluation of multiple histological sections of the spheroids.

Initially, the aim of this study was to compare the radiation sensitivity measured in the 3D assay with the conventional 2D CFA in primary GBM. However, the 2D CFA evaluation was difficult due to the heterogeneous and spreading growth pattern and overlapping colonies of the primary cell lines. Furthermore, the 2D CFAs could not be fitted to the linear–quadratic model due to negative beta terms. In contrast to the 2D CFA, the survival data derived from the 3D assay could be fitted with the LQM and revealed positive beta terms.

In comparison to the 2D assay, the 3D assay has the advantage of being evaluated longitudinally at different time points without fixation. Additionally, the spheroids could also be used for histological analysis. The Coseedis^TM^ 3D culture offers a simple method for the evaluation of radiosensitivity with high reproducibility. A volumetric analysis of the spheroids over time showed a dose-dependent decrease in the volume, which could also be a marker for radiation response. The primary GBM cell line, H74, exhibited a smaller volume in all radiation doses compared to H75 and H77, indicating that the primary cells show patient-specific differences in the volume of the spheroids.

Although the evaluation of the 2D CFA was difficult, both 2D and 3D assays demonstrated a similar radiation response for the three primary GBM cell lines. Gomez-Roman et al. [40] also discovered similar radiosensitivity in 2D and 3D (Alvetex scaffold) culture systems of patient-derived GBM cells. They concluded that the 3D model could simulate the response to radiation, drugs, and molecular-targeted therapies and predict the clinical efficacy of GBM clinical trials.

Jiguet Jiglaire et al. [16] cultured primary GBM cells in a 3D hydrogel model and investigated the radioresponse by MTT Assay. They figured out that most of their primary GBM cultures were more resistant to radiation in this 3D model compared to the classical 2D cultivation. In contrast to the non-adhesive Coseedis^TM^ 3D culture model used in our study, the hydrogel constitutes an extracellular matrix and enables the interaction between cells and the surrounding matrix. This cell–matrix interaction might explain the enhanced radioresistance in this 3D model.

Similar discoveries have been made for other cancer types. The study of Koch et al. [41] demonstrated higher radio- and chemotherapeutic resistance in 3D cultures compared to 2D cultures for colorectal cancer cells. Similar data in terms of chemotherapy resistance in 3D models for breast cancer [42], prostate cancer [43], and ovarian cancer [44] are described. A main reason for the higher drug resistance in 3D models could be the limited access to the drug supply in the center of the spheroid [45]. Further, a radioprotective role of VEGF/Akt signaling was specifically observed in a 3D GBM culture model but not in conventional 2D cultures, emphasizing the importance of preclinical testing of new treatments for GBM in the more representative 3D models [46].

γH2AX IHC staining of the patient-derived GBM spheroids 1 h after irradiation revealed an increase in DNA double-strand breaks after 4 Gy and a further increase after 8 Gy (Figure 6a). After 24 h, the primary GBMs, H75 and H77, showed a reduction in γH2AX-positive cells indicating recovery from DNA damage due to active repair mechanisms. At this time point, we observed significant differences in γH2AX-positive cells in irradiated compared to non-irradiated H74 spheroids suggesting a reduced ability for active DNA repair mechanisms [47]. The histological images showed enhanced γH2AX staining in the center of the spheroids (Figure 6b). The study of Riffle et al. [48] investigated hypoxia, DNA damage, and proliferation in Ewing sarcoma spheroids. They found an increased γH2AX staining in the hypoxic and peri-necrotic regions of the spheroids. In line with our data, they observed Ki67-positive cells mainly in the periphery.

Overall, the 3D culture system used can offer more natural conditions and a cellular environment to study cancer biology in patient-derived glioblastoma. In the future, it might provide a valuable tool to predict patients’ responses to radiation and other therapies.

## 5. Conclusions

This study reports the establishment of a 3D culture model to analyze the radioresponse and characterize patient-derived GBM cells. The agarose-based 3D model provides a good alternative method to analyze the radioresponse of primary GBM cell lines. Especially for GBM cell lines that are difficult to analyze in the standard 2D CFA, the 3D model offers a more reliable method. Moreover, the 3D assay better recapitulates the 3D structure of a tumor, and it also provides the possibility of additional evaluations, such as measurement of the volume and histological analysis to show the 3D architecture of the spheroids. Furthermore, subsequent immunohistological staining of the spheroids can visualize, for example, the distribution of irradiation-induced DNA double-strand breaks.

In summary, this 3D model may better reflect the heterogeneity and radiation response of primary GBM cells than currently available in vitro 2D models and may have a benefit for therapy prediction in personalized medicine.

## Figures and Tables

**Table 1:** Clinical data of the GBM patients from whom the primary cell lines were obtained.

	H74	H75	H77
Brain region	occipital left	parietal left	occipital right
Primary/recurrence	primary	primary	primary
Sex	male	male	male
Age at surgery	62	73	89
Survival after surgery	8 months	35 months	3.5 months
WHO grading	4	4	4
IDH mutation	wildtype	wildtype	wildtype
MGMT	methylated (66%)	methylated (18%)	not methylated (0%)
Nuclear ATRX	retained	retained	retained
p53 mutation	accumulation	no accumulation	accumulation
EGFR	positive	inhomogeneous	inhomogeneous
Ki67 proliferation index	60%	30%	50%

**Table 2:** Radiobiological parameters: D_50_, dose [Gy] to reduce survival fraction to 50%; D_10_, dose [Gy] to reduce survival fraction to 10%. α-values and β-values were derived from the linear quadratic model: SF = exp^(−αD−βD²)^. The survival fraction SF is described by the radiation dose D, the linear coefficient α, and the quadratic coefficient β.

	H74	H75	H77
α [Gy^−1^]	0.3671	0.2167	0.1398
β [Gy^−2^]	0.0103	0.0144	0.0338
D_50_ [Gy]	1.79	2.69	2.90
D_10_ [Gy]	5.42	7.20	6.41

## Data Availability

Data are contained within the article.
